# Measured Copper Toxicity to *Cnesterodon decemmaculatus* (Pisces: Poeciliidae) and Predicted by Biotic Ligand Model in Pilcomayo River Water: A Step for a Cross-Fish-Species Extrapolation

**DOI:** 10.1155/2012/849315

**Published:** 2012-03-05

**Authors:** María Victoria Casares, Laura I. de Cabo, Rafael S. Seoane, Oscar E. Natale, Milagros Castro Ríos, Cristian Weigandt, Alicia F. de Iorio

**Affiliations:** ^1^Bernardino Rivadavia National Museum of Natural History, Avenida Angel Gallardo 470, C1405DJR Buenos Aires, Argentina; ^2^National Water Institute, Autopista Ezeiza-Cañuelas, Tramo Jorge Newbery km 1.62 (1802), Ezeiza, C1004AA1 Buenos Aires, Argentina; ^3^Faculty of Engineering, University of Buenos Aires, Avenida Las Heras 2214, C1127AAR Buenos Aires, Argentina; ^4^Faculty of Agronomy, University of Buenos Aires, Avenida San Martín 4453, C1417DSE Buenos Aires, Argentina

## Abstract

In order to determine copper toxicity (LC50) to a local species (*Cnesterodon decemmaculatus*) in the South American Pilcomayo River water and evaluate a cross-fish-species extrapolation of Biotic Ligand Model, a 96 h acute copper toxicity test was performed. The dissolved copper concentrations tested were 0.05, 0.19, 0.39, 0.61, 0.73, 1.01, and 1.42 mg Cu L^−1^. The 96 h Cu LC50 calculated was 0.655 mg L^−1^ (0.823 − 0.488). 96-h Cu LC50 predicted by BLM for *Pimephales promelas* was 0.722 mg L^−1^. Analysis of the inter-seasonal variation of the main water quality parameters indicates that a higher protective effect of calcium, magnesium, sodium, sulphate, and chloride is expected during the dry season. The very high load of total suspended solids in this river might be a key factor in determining copper distribution between solid and solution phases. A cross-fish-species extrapolation of copper BLM is valid within the water quality parameters and experimental conditions of this toxicity test.

## 1. Introduction

The number of large-scale mining operations has been increasing greatly in Argentina during the last decade. It has resulted in social and environmental conflicts of diverse scale [1]. Some river basins are seriously polluted by heavy metals released by present and ancient mining activities [2]. Furthermore, occasional accidents have aggravated this situation by suddenly introducing substantial amounts of heavy metals into aquatic environments, which might be accompanied by changes in water pH, depending on the type of the mining effluent in question. Some tributaries to the upper Pilcomayo River, in Bolivia, drain a large conical peak known as Cerro Rico de Potosí. This mountain is partially composed of precious metal-polymetallic tin ores. Mining of Potosí ores began in 1545 and has led to the severe contamination of the Pilcomayo River water and sediments. Although toxic waste spills are released daily in the upper basin of the Pilcomayo River, in 1996 and 2005 mine tailings dams collapsed and thousands of tons of toxic wastes have been spread downstream. These toxic spills, which contain high concentrations of arsenic and heavy metals, may severely affect plants, animals, and human health, even several kilometers downstream. For example, in Spain, the Aznalcóllar accident (1998) has severely contaminated the Guadiamar River [3] and the accident at the El Porco mine in Bolivia (in 1996, 50 km from the city of Potosí) contaminated the Pilaya River and part of the Pilcomayo River [4].

Copper is one of the most abundant heavy metals present in the Pilcomayo River water and sediments [5]. Copper is a trace element which is essential to the function of specific proteins and enzymes. However, at high concentrations, it may be toxic to organisms. The toxicity of copper to fish has been well documented. In addition to its acute lethality, a wide range of toxicological responses of several organs to this metal has been reported for various fish species. Copper alters the regular functioning of the gills and liver [6, 7] by causing severe histological changes in these organs. The most frequent physiological effect observed in fish exposed to aqueous copper is ionoregulatory failure [6]. Additionally, aqueous copper has also been reported to influence fish respiration [8, 9]. Biota differences in respiratory physiology, including differences in ventilation rates and volumes, can lead to different internal exposure doses and thus different toxic responses [10]. These differences in respiratory responses to a pollutant might be important.

The impact of copper on the aquatic environment is complex and depends on the physicochemical characteristics of water. Alkalinity, hardness, dissolved organic matter, and pH strongly influence copper speciation in water and, consequently, its bioavailability for absorption by fish [11]. Ionic copper (Cu^2+^) and copper hydroxides are considered the most toxic species of aqueous copper, while copper carbonates have proven much less toxicity [12]. Cu^2+^ is the dominant copper species at pH levels below 7.0, and according to the mean lethal concentration (LC50) ranges in [13] copper is the second most toxic metal to freshwater fish. In soft water, copper is acutely toxic to freshwater teleosts at concentrations between 10 and 20 *μ*g L^−1^ [14–16] including such cultured species as salmonids, cyprinids, and catfish [17]. Ion-poor and soft waters [18] have a low buffering capacity, and fish culture practices may be accompanied by changes in pH and, hence, in the chemical speciation of copper, potentially increasing its toxic effect. Furthermore, high temperatures tend to increase the diffusion rate, accelerating chemical reactions [19], thereby favoring the toxic action of copper or other heavy metals.

The Pilcomayo River water is characterized by its high concentrations of calcium, sodium, (bi)carbonate, sulphates, and total suspended solids. Water hardness is one of the main and most well recognized of the modifying factors of metal ionic species. Hardness reduces toxicity by “protecting” the organism against metal toxicity via several possible mechanisms [20, 21]. The ameliorative effect of hardness is shown to be more complex than the simple hardness-toxicity relationships would suggest. The hardness cations, calcium and/or magnesium, and protons are thought to inhibit Cu binding/uptake at the cell surface, via different mechanisms [22]. Alkalinity, on the other hand, affects metal ionic species in water solution through their complexation with carbonates [23, 24]. Additionally, dissolved organic matter binds metal species as well [25].

The conceptual Biotic Ligand Model (BLM) [26] may be considered in terms of three separate components: water chemistry, the binding of the toxic metal species to the biotic ligand, and the relationship between metal binding and the toxic response of the aquatic organism [27]. The BLM has been proposed as a tool to evaluate quantitatively the manner in which water chemistry affects the speciation and biological availability of metals in aquatic systems [27]. The toxicology of metals would not be complete without an evaluation of which chemical species are the most toxic and how toxicity might be modified by various environmental factors. These mechanisms need to be uncoupled, if their effects are to be incorporated into models such as the BLM [22], which uses physicochemical variables to predict the acute toxicity of metals, such as copper, to freshwater biota on a site-specific basis. At present, BLM, version 2.2.3, has been developed for two species of fish: fathead minnows (*Pimephales promelas*) and rainbow trout (*Oncorhynchus mykiss*), for three species of invertebrates: *Daphnia magna, Daphnia pulex, *and* Ceriodaphnia dubia* and four metals: copper, cadmium, silver, and zinc. BLM-predicted Cu LC50 values have agreed to estimated LC50 values over a wide range of water quality characteristics [27]. It is implicitly assumed that BLMs can be extrapolated within taxonomically similar groups; that is, BLMs developed for *P. promelas *can be applied to toxicity data for other fish species, and BLMs for *D. magna* and *C. dubia* can be applied to toxicity data for other invertebrates [28]. The basis for a cross-species extrapolation is the assumption that the parameters which describe interactions between cations (notably calcium, magnesium, and protons), the toxic free metal ion (e.g., Cu^2+^), and the biotic ligands are similar across organisms and that only intrinsic sensitivity varies among species [28].

*Daphnia magna* acute toxicity tests have been performed in the Pilcomayo River water from Mision La Paz, Argentina, by Natale et al. [29]. But there are no previous toxicity tests on a vertebrate in the Pilcomayo River water. *Cnesterodon decemmaculatus* (Pisces: Poeciliidae; Jenyns, 1842) is an endemic member of the fish family Poeciliidae with extensive distribution in Neotropical America. The species attains high densities in a large variety of water bodies within the entire La Plata River and other South American basins. *Cnesterodon decemmaculatus* is a small, viviparous, microomnivorous, benthic-pelagic, nonmigratory fish (maxi-minimum size, *≈*25 and 45 mm for ♂♂ and ♀♀, resp.). This species is easy to handle and breed under laboratory conditions. Also, *C. decemmaculatus* proved to be adequate as test organism, due to its small size, fast growth, and short reproduction period [30]. Furthermore, several reports found this species to be suitable as a test organism in acute and chronic toxicity bioassays. The ranges of tolerance of *C. decemmaculatus* to many environmental parameters, for example, temperature, salinity, and pH, match the conditions for most toxicity tests. *Cnesterodon decemmaculatus* is usually found in anoxic or very scarcely oxygenated water bodies as well. Thereby, *C. decemmaculatus* has been used by several authors in bioassays [31–35]. *Pimephales promelas *(Pisces: Cyprinidae; Rafinesque, 1820), one of the fish species for which BLM has been developed, is a temperate, holarctic fresh water fish. As well as *C. decemmaculatus*, it is quite tolerant to turbid, low-oxygenated water bodies and can be found in muddy ponds and streams that might, otherwise, be inhospitable to other species of fish. It can also be found in small rivers. Because of its relative resilience and large number of offspring produced, US EPA guidelines (United States Environmental Protection Agency) outline its use for the evaluation of acute and chronic toxicity of water samples or chemical species in vertebrate aquatic animals [36, 37].

The aims of this study were to (a) assess Cu toxicity (96 h LC50) to *C. decemmaculatus* in a surface water with high hardness, sodium, sulfate, and chloride concentrations (Pilcomayo River water), (b) apply BLM, version 2.2.3, to predict acute copper toxicity to *P. promelas* (Cu LC50) under Pilcomayo River water characteristics, (c) compare the predicted Cu LC50 value for *P. promelas* to the calculated for* C. decemmaculatus *in the Pilcomayo River water, and, finally, (d) given that Pilcomayo River hydrochemistry is strongly influenced by the hydrological cycle [34], we also analyze the interseasonal variation of the main water quality parameters that influence copper bioavailability and toxicity.

## 2. Materials and Methods

### 2.1. Study Area

**Figure 1:**
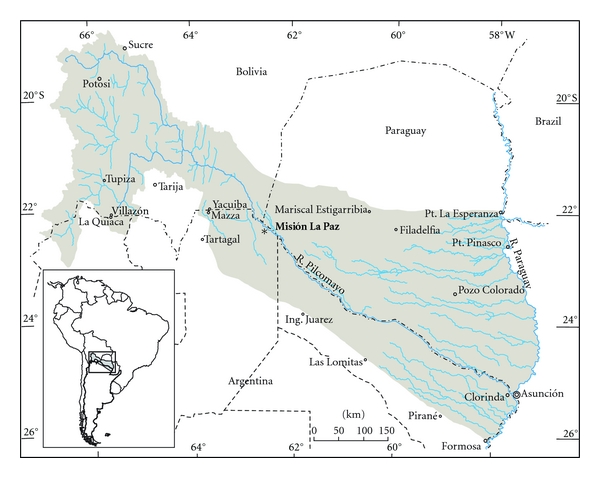
Map of the Pilcomayo River basin with the water sampling location (Misión La Paz, Argentina).

The Pilcomayo River in South America is a tributary to the large La Plata system. Its headwaters are located in Bolivia along the eastern flank of the Central Andes at an elevation of approximately 5,200 m (Figure 1). The river flows in a southeasterly direction for about 670 km until reaching the Chaco Plains along Bolivia's southern border with Argentina. Its total length is 2,426 km, and its basin covers an area of approximately 288,360 km^2^ (Comisión Trinacional del Río Pilcomayo).

An important feature of the Pilcomayo River, present in all dryland rivers, is its extreme interannual and interseasonal variability in discharge [38]. Interseasonal climatic variation is also extreme in dryland river basins as, frequently, a clearly marked dry and rainy season can be distinguished. These different hydrological regimes are usually associated with important variations in water chemistry and may have important effects on the behavior of aquatic ecosystems and trace metals toxicity.

### 2.2. Water Sampling and Chemical Analysis

 Discrete water samples for chemical analyses were taken 10 cm below the water surface and in triplicate from the navigation channel, left and right shore of the Pilcomayo River in the Misión La Paz International bridge (22°22′45′′ S–62°31′08′′ W; 254 meters over sea level) in May 2009 (Figure 1). Water sampling took place during the routine water quality monitoring program coordinated by the Subsecretaría de Recursos Hídricos (SsRH-Argentina) and the Comisión Trinacional del Río Pilcomayo. Sampling and in situ water quality determinations were in charge of the SsRH, Centro de Ecología Aplicada del Litoral (CECOAL-CONICET) and Universidad Nacional de Salta (UNS). Laboratory analysis of chemical parameters was performed by the UNS and the Comisión Nacional de Energía Atómica (CNEA-Argentina). Water discharge (Q) was measured by EVARSA-Argentina, pH, and water temperature (T) were determined in situ. Dissolved concentrations of calcium (Ca), magnesium (Mg), chloride (Cl), potassium (K), sodium (Na), sulphate (SO_4_), alkalinity (Alk), dissolved organic carbon (DOC), total suspended solids (TSS), total dissolved solids (TDS), and total (T·Cu) and dissolved copper (D·Cu) concentrations were determined using Standard Methods test protocols [39]. Particulate copper (P·Cu) was derived according to the following equation:

(1) $$\text{P}\cdot \text{Cu}=\frac{\left[\text{T}\cdot \text{Cu}\right]-\left[\text{D}\cdot \text{Cu}\right]}{\left[\text{TSS}\right]}.$$

### 2.3. Toxicity Test

Water for the toxicity test was collected in prerinsed 10 L polypropylene containers. Samples were immediately placed into coolers and transported to the laboratory. Later, water was centrifuged (2,000 rpm during 15 minutes) and filtered through 47 mm 0.45 *μ*m pore glass-fiber filters (Whatman GF/C). Copper background concentration in the Pilcomayo river water was 0.02 mg Cu L^−1^.

Juvenile *C. decemmaculatus* were collected from a small pond, located in Reserva Natural Los Robles, Buenos Aires Province, Argentina (main chemical and physical parameters are shown in Table 2). Fish were kept at temperatures ranging from 20 to 24°C and pH ranging from pH 7.1 to 7.5 in an aquarium supplied with a continuous flow of aerated de-chlorinated tap water for 30 days. During this period and posterior laboratory and test water (centrifuged and filtered Pilcomayo River water) acclimation, the fish were fed with a daily ration of commercial fish food Shulet. Acclimation to test water (pH of 7.9–8.20, 15–20°C) was performed by adding small quantities of test water to the aquarium until most of the water volume corresponded to test water. One day before and during the experiment, fish were not fed.

Toxicity effect of copper on fish was tested in static systems (4 L glass aquaria) with continuous artificial aeration, constant environmental temperature (20°C), and natural laboratory photoperiod. Test water volume in each aquarium was 2 L. The experimental design included seven different copper concentrations with one control group (kept in test water and without copper addition). Test copper concentrations were attained by spiking from a stock solution of 100 mg Cu L^−1^. The toxicant used was reagent-grade CuSO_4_. Dissolved copper concentrations tested were 0.05, 0.19, 0.39, 0.61, 0.73, 1.01, and 1.42 mg Cu L^−1^. 

To define the range of copper concentrations to be employed in the bioassay, a nominal concentration of 0.8 mg Cu L^−1^ was tested in an aquarium with 2 L volume of the Pilcomayo River water and 12 acclimated specimens of juvenile *C. decemmaculatus* for 96 h. Fish (not sexed) taken from the acclimation tank were randomly distributed in the different experimental aquaria. Mean standard length of the specimens selected was 18.9 mm, and each aquarium contained 10 specimens. Copper concentration in the experimental aquaria was adjusted prior to the fish transfer. Survival was registered four times a day during 96 h. Water pH, conductivity, and dissolved oxygen were measured with portable probes from HANNA (HANNA instruments, Inc. Woonsocket, RI, USA) daily. Water samples were collected into polypropylene conical tubes and acidified to pH < 2 with concentrated nitric acid (reagent grade) for metal analysis by atomic absorption spectrophotometry (Perkin Elmer 1100B, Perkin Elmer, Inc., Waltham, MA, USA) after acid digestion (HNO_3_:HClO_4_:HF:HCl). Method copper detection limit was 0.01 mg L^−1^. 

### 2.4. LC50 Calculations

The median lethal concentrations (LC50) at 24, 48, 72, and 96 h (24 h LC50, 48 h LC50, 72 h LC50, 96 h LC50) were calculated using the PROBIT method [40] and the statistical program Statgraphics Plus 5.1 (StatPoint Technologies, Inc., Warrenton, VI, USA). 

Version 2.2.3 of the BLM Windows Interface (available at http://www.hydroqual.com/wr_blm.html) was run in order to predict acute copper toxicity to *P. promelas* (toxicity mode) and copper ionic speciation (speciation mode) on the measured copper concentrations tested. The Pilcomayo River water quality parameters employed to run the BLM were temperature, pH, dissolved organic carbon, calcium, magnesium, sodium, potassium, sulphates, chlorides, alkalinity, and dissolved copper concentrations. 

### 2.5. Interseasonal Water Quality Analysis

To determine water discharge influence on major anions and cations and on total and dissolved solids and copper concentrations, we used water quality data, available from 2003 to 2010, from the Misión La Paz monitoring station (provided by La Comisión Trinacional del Río Pilcomayo). Though a large number of water quality parameters are determined, we selected only those that constitute BLM inputs, water discharge, total suspended and dissolved solids, and total and dissolved copper concentrations. Hydrological and water quality data were classified into two groups: data corresponding to the dry season (May–October) and data corresponding to the wet season (November–April). Distance metric test statistic (*dm*) [41, 42] was calculated in order to determine significant difference between the means. This statistic is defined as the difference between the variables means *x* and *y* of the standardized series. Due to the limited data availability for each season, the standard deviations of the errors, ES*_x_* and ES*_y_*, were estimated using bootstrap techniques [43]. Bootstrapping is the practice of estimating properties of an estimator (standard deviations of the errors, in this case) by measuring those properties when sampling from an approximate distribution. It can be implemented by constructing a number of re-samples of the observed dataset (and of equal size to the observed dataset). Each re-sample is obtained by random sampling with replacement from the original dataset. 

The *dm* statistic is defined as follows:

(2) $$dm=\frac{\bar{x}-\bar{y}}{\sqrt{\text{E}{{\text{S}}_{x}}^{2}+\text{E}{{\text{S}}_{y}}^{2}}},$$

where values of |*dm*| higher than 2 are an indication that the corresponding variables means are different. 

Pilcomayo River water discharge available data from 1961 to 2008 (provided by SsRH-Argentina) was used to perform the Pilcomayo River hydrograph. 

## 3. Results

### 3.1. Toxicity Test

**Figure 2:**
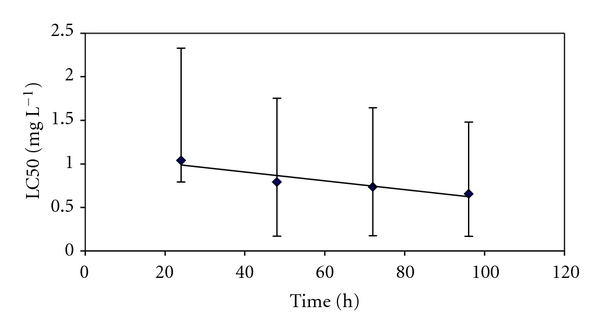
*Cnesterodon decemmaculatus* copper toxicity test: Cu LC50 values (mg L^−1^) calculated by PROBIT analysis and confidence intervals (vertical bars) as a function of exposure time (h).

No mortality was observed in the control group. An exponential decrease of fish survival with time towards an asymptotic value reached at about 96 h was observed.  Figure 2 shows LC50 values as a function of copper exposure time. Data fitted an exponential regression leading to the following equation: LC50 = 1.1428*e* ^−0.0061t^ and a *R* ^2^ value of 0.9219. 

The median lethal concentrations (LC50, mg L^−1^) at 24, 48, 72, and 96 h (24 h LC50, 48 h LC50, 72 h LC50, 96 h LC50) with their corresponding confidence intervals (quoted) calculated using PROBIT method were 1.039 (1.288–0.245), 0.792 (0.962–0.622), 0.734 (0.908–0.561), and 0.655 (0.823–0.488), respectively (Figure 2). 

### 3.2. Biotic Ligand Model

**Figure 3:**
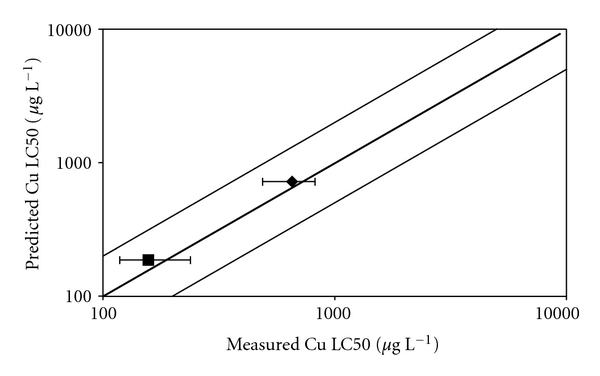
Measured copper toxicity (LC50, in *μ*g L^−1^) to *C. decemmaculatus* by Villar et al. (closed square) and the present study (closed diamond) compared with predicted copper toxicity using the BLM developed for *P. promelas*. The thicker line represents a 1 : 1 relationship. The thinner line represents predictions within a factor of 2. The error bars represent 95% confidence intervals.

All physicochemical parameters values of the Pilcomayo River water, measured on our sampling date (Table 2), were within the range to which BLM can be applied. Calculated 96 h Cu LC50 for *C. decemmaculatus* was 0.655 mg L^−1^ (0.823–0.488). Predicted 96 h Cu LC50 by BLM developed for *P. promelas* was 0.722 mg L^−1^. BLM was also run with water quality data of the test water used by Villar et al. [44] in order to obtain a predicted acute copper toxicity concentration in a soft water.  Figure 3 shows that predicted Cu LC50 (*μ*g L^−1^) was accurate within a factor of 2 for both, hard and soft water (the Pilcomayo River water quality data and Villar et al. [44]). 

**Figure 4:**
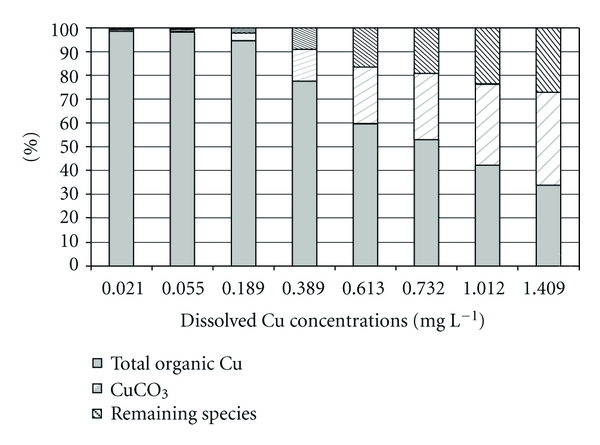
BLM speciation output for each of the copper concentrations tested and control group (first column). Copper species are expressed as percentages of total dissolved copper concentration. Remaining species summarizes the contributions of CuOH, Cu(OH)_2_, CuSO_4_, Cu(CO_3_)_2_^−2^, CuCl^+^, and CuHCO_3_^+^.

BLM copper speciation (Figure 4) shows that CuCO_3_ is the second most abundant copper chemical species in the control group, in all the concentrations tested up to 0.732 mg L^−1^ and becomes the most abundant in the last two concentrations, after copper bound to dissolved organic carbon. CuHCO_3_^+^ contribution, amongst the remaining species, is the highest and reaches 27% for the highest copper concentration tested. Moreover, for these two cases, the carbonate fraction of dissolved copper exceeds the organic fraction. 

### 3.3. Interseasonal Water Quality Analysis

Means, medians, standard deviations, maximum, and minimum values of the selected water quality parameters are shown in Table 1. Water quality of the Pilcomayo River water sampled to perform the bioassay and BLM modeling corresponded to the dry season. For this water sample, pH, TDS, Ca, Mg, Na, K, SO_4_, Cl, and Alk were lower than the median values from historical records of the Pilcomayo River in the dry season. However, the respective comparison for water discharge and total suspended solids concentration showed a reverse outcome. 

According to *dm* values (Table 2), it can be seen that water discharge, temperature, calcium, magnesium, sulphates, chlorides, sodium, total suspended and dissolved solids, and total copper concentrations showed interseasonal variation. Although all *dm* values for these variables are higher than 2, the value itself shows how different the corresponding means are. Total suspended solids and total copper concentrations show interseasonal variation, but the difference between means (*dm*) is lower compared to other water quality variables. Alkalinity, pH, potassium, particulate copper, and dissolved copper concentrations do not show interseasonal variation (*dm* values lower than 2). According to *dm* values, water discharge, temperature, total suspended solids, and total copper are higher during the wet season. The remaining water quality parameters show higher concentrations during the dry season. 

**Figure 5:**
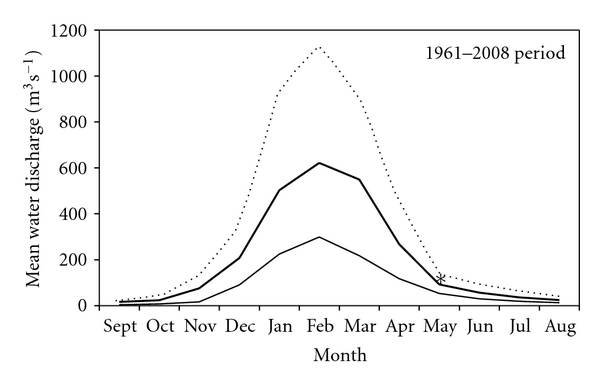
The Pilcomayo River hydrograph. The thicker highlighted line represents mean river water discharge. The dotted line represents the 90th percentile water discharge values and the lower thinner line the 10th percentile water discharge values. The asterisk indicates mean water discharge on May 2009 (water sampling), 123.6 m^3^ s^−1^. (Data from Misión La Paz monitoring station, Argentina.)

The Pilcomayo River hydrograph (Figure 5) is typical of dryland rivers. Water discharge begins to increase on November, peaks on February, and declines gradually reaching the lowest values on September. Mean annual water discharge determined with water discharge record of the last 47 years at Misión La Paz was 212.1 m^3^ s^−1^ with a maximum value of 508.7 m^3^ s^−1^ and a minimum of 77.7 m^3^ s^−1^. The Pilcomayo River maximum discharge record was registered on March 1984 when water discharge tripled its mean value reaching 1908 m^3^ s^−1^. The corresponding minimum water discharge value of 7.5 m^3^ s^−1^ was registered on September 1966. The mean Pilcomayo River water discharge on May 2009, our sampling date (123.6 m^3^ s^−1^), was higher than the historical mean water discharge for May (98.8 m^3^ s^−1^, data not shown) and corresponded to the 75th percentile.

## 4. Discussion

The Pilcomayo River water is very hard surface water. Water hardness is mainly produced by calcium and magnesium concentrations [39]. Copper toxicity to several aquatic species has been reported to be negatively correlated with hardness, but other reports have indicated little or no effect [23]. The effect of hardness on copper toxicity might reflect competition between hardness ions and copper for binding sites on gill surface. Calcium appears to be more protective than magnesium against copper toxicity to fish [45]. Calcium binds to the gill surface and controls the permeability of the membrane and the integrity of the ionoregulatory function [46]. There is only one previous measure of copper toxicity to *C. decemmaculatus*. Villar et al. [44] found for adults of *C. decemmaculatus* a 96 h Cu LC50 of 0.155 mg L^−1^ in a synthetic soft water with a hardness of 67.66 mg CaCO_3_ L^−1^. Acute copper toxicity estimates from this study and Villar et al. [44] were normalized to a hardness of 50 mg CaCO_3_ L^−1^ using the US EPA conversion formula for normalization of data given in the ambient water quality criteria for copper (LC50 at 50 mg/L = e^ln⁡(LC50) − 0.9422 × (ln⁡(hardness) − ln⁡(50))^) [47]. Normalization of this study toxicity estimates gave a LC50 of 0.12 mg L^−1^ (0.08–0.15), and for Villar et al. [44] the normalized LC50 was 0.12 mg L^−1^ (0.09–0.18). Although test water used by Villar et al. [44] had lower dissolved organic carbon and alkalinity concentrations, hardness seems to have a strong influence on copper toxicity to *C. decemmaculatus*. Van Genderen et al. [45] found that increments in water hardness from 200 to 1000 mg CaCO_3_ L^−1^, achieved by increasing concentration of calcium (magnesium held constant at 30 mg L^−1^), increased 96 h LC50 to larval *P. promelas*. Some studies have suggested that the molar ratio between calcium and magnesium may be more important than their absolute concentrations. The calcium-to-magnesium molar ratio in the Pilcomayo River water is 1.45, and studies reported that hardness consisting primarily of calcium (molar ratios of >1) is protective of both fish [19, 23] and invertebrates [45]. However, hardness consisting primarily of magnesium (Ca : Mg molar ratios of ≤1) has only been shown to be important for invertebrates [22]. 

Alkalinity affects copper speciation in solution through complexation with carbonates, which will influence bioavailability [22]. The effects of hardness on aquatic biota toxicity due to metals in some cases are misinterpreted by correlations with alkalinity, pH, and/or other ionic constituents. Van Genderen et al. [45] found that the relationship between alkalinity and LC50 values for *P. promelas *in the natural waters tested was not significant, but analysis of laboratory water quality data demonstrated a significant positive correlation. Lauren and MacDonald [24] similarly concluded that cupric ion and copper hydroxo complexes, but not copper carbonate complexes, were toxic. When alkalinity is increased, while maintaining a constant pH, copper toxicity has been reported to decrease, but the magnitude of this effect varies with hardness and other experimental conditions and is sometimes not observed [23]. These results show the strong influence of alkalinity on copper bioavailability as copper concentration increases. 

In the present study, pH varied from 7.90 to 8.30 in all treatments. Erickson et al. [23] found a decrease in copper toxicity to early-life-stage *P. promelas* when pH was increased from 6.5 to 8.5–9 in ambient alkalinity (45 mg CaCO_3_ L^−1^) as well as in elevated alkalinity (150 mg CaCO_3_ L^−1^). On the other hand, Lauren and MacDonald [24] concluded that alkalinity, but not pH, affected short-term lethality of copper to rainbow trout. Carvalho and Fernandes [19] found that copper toxicity to *Prochilodus scrofa* is dependent on water pH. They found lower copper toxicity at pH of 4.5. The stimulation, at low pH, of gill secretion of mucus, which can bind copper, might contribute to the antagonism of low pH with copper toxicity [23]. To perform their toxicity tests, these authors used soft low-alkalinity water and is possible that the reduced concentration of protons and the low levels of calcium in ion-poor soft waters may favor Cu^2+^ binding to the gill surface membrane, increasing the uptake of copper and, hence, its toxicity in high water pH [12]. 

Erickson et al. [23] found that the addition of potassium chloride increased copper toxicity, while addition of calcium chloride and sodium chloride reduced it, and magnesium chloride had no effect. When calcium, sodium, and magnesium were added as sulfate salts, the same effects were observed. The primary effect of copper is on sodium and chloride uptake and efflux [48]. Exposure to copper produces the inhibition of the active uptake of sodium. In addition, at high enough concentrations, it may also affect the efflux, but this effect would mainly be mediated by a general disruption in gill epithelial integrity. The effects on ionoregulation result in a decrease in levels of plasma sodium, chloride, and other ions, which in turn leads to cardiovascular collapse and death. There is the hypothesis that Cu^2+^ is reduced to Cu^+^ by reductases on the cell surface to facilitate uptake via sodium transporters [22]; this might explain the reason why the addition of external sodium and chloride is expected to reduce copper toxicity. It is possible that high sodium and chloride concentrations found in the Pilcomayo River water have some protective effect against copper toxicity. Chloride may also have an effect on copper speciation. However, in BLM copper speciation output (Figure 4), copper chloride is included within the remaining species and its contribution is the lowest. Consequently, in this study, chloride effect on copper bioavailability is negligible. 

Other water quality parameters that are considered for their ability to ameliorate copper toxicity by decreasing copper bioavailability are the complexing ligands (dissolved organic carbon, hydroxide, and carbonate) [42]. Matsuo et al. [25] showed that dissolved organic matter forms complexes with Cu^2+^, which reduces the free form in water and therefore the amount of ionic Cu^2+^ available to bind to the gill sites. These authors concluded that dissolved organic matter has direct effects on the gills because it complexes Cu^2+^ and acts on the transport and permeability properties of the gills. Tao et al. [49] proposed that organic compounds with metals bound may adhere to the mucus of the epithelial cell surface during fish aspiration, and afterwards the dissociation of the complex could then release free copper which, in turn, could be transported into the gill tissue. However, the uptake rate of these compounds would be much slower when compared with that of free ionic copper. In the Pilcomayo River water, the effect of dissolved organic matter (measured as dissolved organic carbon) on copper bioavailability is evident. Organic copper is the most abundant species in all treatments except in the highest copper concentration. Paquin et al. [27] argued that strong ligands, such as dissolved organic matter, at the metal concentrations used in acute toxicity applications could reach saturation and do not exert a controlling influence over metal speciation. Bryan et al. [50] found a higher complexation of copper by dissolved organic matter at low total copper concentrations. They also found that, in the absence of dissolved organic matter and at pH of 8.5, complexation by carbonate species is considerable, but where the complex CuCO_3_, rather than CuHCO_3_^+^, is dominant. In our study, BLM shows that there is a reduction in the percentage of copper bound to organic matter and an increment in CuCO_3_ and secondary in CuHCO_3_^+^, as copper concentration increases. 

Few studies have examined the effects of suspended solids on copper toxicity. Erickson et al. [23] results suggested that copper adsorbed onto suspended solids could not be considered to be strictly nontoxic. Tao et al. [51] proposed a mechanism of particulate metal uptake by fish, by desorption of the metal from the particles within the gill microenvironment where the particles adhered to mucus. Natale et al. [29] found higher copper toxicity to *D. magna* in unfiltered Pilcomayo River water samples compared to toxicity test performed with filtered Pilcomayo River water. The authors attributed the difference to the presence of the suspended solids themselves and/or to bioavailability of toxicants (i.e., copper and other metals) adsorbed onto the particles. To avoid these effects of total suspended solids and for the purpose of comparing the experimental results of the copper toxicity bioassay with the corresponding BLM (which considers that metal bound to particulate matter exerts no toxicity) estimates, test water in our study was centrifuged and filtered. Consequently, the experimental approach employed in this study was not able to provide evidence on the effects of total suspended solids on copper toxicity. If copper bound to total suspended solids is nontoxic to fish species, toxicity measured on the basis of dissolved copper in tests performed with unfiltered Pilcomayo River water should not differ from our results. 

In a dryland river basin, as Pilcomayo River, it is expected that differences in water discharge values between the dry and the wet season would influence dissolved concentrations of the water quality variables that determine copper bioavailability and toxicity. During the dry season, higher dissolved calcium, magnesium, sodium, and chloride concentrations may reduce copper toxicity to fish while the opposite is expected during the wet season when dissolved concentrations decrease by effect of dilution. Our water sampling was performed at the onset of the dry season, when dissolved concentrations of major ions begin to increase. Therefore, a higher protective effect of these ions should be observed in a study conducted in the Pilcomayo River water collected at lower water discharges. During the dry season, water hardness can reach values between 400 and 500 mg CaCO_3_ L^−1^ however, its median value is 332.5 mg CaCO_3_ L^−1^. Even though BLM was developed from tests generated in soft and moderately hard waters (≤250 mg CaCO_3_ L^−1^), previous studies have suggested that BLM predictions are still accurate in very hard surface waters [45]. During the wet season, water hardness falls to a median value of 184.6 mg CaCO_3_ L^−1^. 

Temporal variation of dissolved organic carbon was impossible to analyze due to lack of historical data, but our results show a possible saturation of dissolved organic carbon binding sites as copper concentration increases, leading to an increment in carbonate species. Alkalinity did not show temporal variation in its concentration. The effect it exerts on copper bioavailability between seasons will depend more on dissolved organic carbon and dissolved copper concentrations than on alkalinity concentration itself. 

 The Pilcomayo River high load of suspended solids originates in the erosion of soils in the upper mountainous region of the basin during the rainy season. When toxic waste spill from mine tailings is released into the river, copper adsorbs onto suspended solids and sediment. During the rainy season, sediments and solids resulting from eroding soils are carried downstream. Table 1 shows that the highest percentage of total copper concentration belongs to copper adsorbed onto suspended solids. Thus, during the dry season, lower water discharges promote sedimentation leading to lower total suspended solids and consequently lower total copper concentrations in the water column. 

Dissolved copper concentration did not show interseasonal variation. This means that the differences in water discharge values between the dry and the wet seasons would not influence dissolved copper concentrations. Also particulate copper concentration did not show temporal variation. Based on the Lu and Allen approach [52], we calculated the partition coefficient (*Kd* = *P* · Cu/*D* · Cu) for each season and the *dm* statistic. According to the *dm* value (lower than 2, data not shown), *Kd* does not show interseasonal variation. The partitioning of copper onto suspended particulate matter of rivers depends on many factors including the solid amount. According to Lu and Allen [52], when total suspended solids are high (100 mg L^−1^), *Kd* can be considered to be independent of copper concentration. These authors also found lower *Kd* values with increasing total suspended solids concentration and that this decrease was less at higher total suspended solids concentration. BLM-MONTE model [53] estimates *Kd*(*Kd* = 1.04 × 10^6^ × TSS^−0.7436^) showing this copper-particulates inverse relationship. Total suspended solids values in the Pilcomayo River are much higher, reaching a value of more than 50,000 mg L^−1^. Therefore, in this extreme case, *Kd* and dissolved copper concentration could be independent of total suspended solids concentration. We could assume that, given that particulate copper showed no variation between the dry and the wet seasons, the copper associated to binding sites is always the same. Attention should be given to the fact that dissolved copper concentrations records in the Pilcomayo River are quite under fish acute toxicity levels along the entire hydrological cycle. 

## 5. Conclusions 

We can conclude that both *P. promelas* and *C. decemmaculatus* fish species respond similarly to copper, and a cross-species extrapolation of Cu BLM is valid within the Pilcomayo River water quality characteristic parameters and experimental conditions of this toxicity test. For a complete cross-fish-species extrapolation, acute copper toxicity tests across a wide range of water quality conditions should be performed to determine if Cu BLM has the ability to account for differences in toxicity to the fish species tested under various site-specific differences in water quality characteristic parameters. 

This study shows the importance of studying temporal variation in water quality variables to derive accurate water quality criteria for toxic metals. In the Pilcomayo River, several water quality parameters related to copper toxicity (calcium, magnesium, sodium, and chlorides) vary significantly from the wet season to the dry season and so the protective effect they can exert on fish. On the other hand, the very high load of suspended solids seems to play an important role in determining copper bioavailability and toxicity, since most of the copper appears adsorbed onto these solids and only a small fraction keeps dissolved and almost invariable between the high and the low water discharge seasons. 

## Figures and Tables

**Table 1:** Main chemical and physical parameters of the water where *C. decemmaculatus* specimens were captured (ND: not detected).

Parameter	
T (°C)	17
pH	7.86
CE (*μ*S)	512
Diss. O_2 _(mg L^−1^)	11.02
NH_4_ (mg L^−1^)	0.005
NO_3_ (mg L^−1^)	0.006
NO_2_ (mg L^−1^)	0.003
SRP (mg L^−1^)	0.141
SO_4_ (mg L^−1^)	14.2
Cl (mg L^−1^)	17.5
Alkalinity (mg CaCO_3_ L^−1^)	292.9
Mg (mg L^−1^)	17.8
Ca (mg L^−1^)	27.7
Cu (mg L^−1^)	ND
Zn (mg L^−1^)	0.03
Cr (mg L^−1^)	0.04
Cd (mg L^−1^)	ND
Pb (mg L^−1^)	0.15

**Table 2:** Seasonal descriptive statistics and *dm* statistic values (difference between season's means) of hydrological and water quality parameters of the Pilcomayo River at Misión La Paz, Argentina (data provided by the Comisión Trinacional del Río Pilcomayo).

		Q	pH	T	TSS	TDS	Ca	Mg	SO_4_	Alk	Cl	Na	K	DOC	T·Cu	P·Cu	D·Cu
		m^ 3^ s^−1^	UpH	°C	mg L^−1^	mg L^−1^	mg L^−1^	mg L^−1^	mg L^−1^	Mg CaCO_3_ L^−1^	mg L^−1^	mg L^−1^	mg L^−1^	mg L^−1^	mg L^−1^	*μ*g kg^−1^	mg L^−1^
Sampling date (May 2009)		75.20	7.67	21.43	1637	517	73.33	30.5	207.3	110	101	65.2	5.1	4.4	0.07	0.07	0.001

Dry season	*n*	14	14	11	13	11	14	14	12	12	12	14	14	6	13	12	14
	Mean	57.18	7.87	17.54	1582.95	845.7	80.6	35.22	292.09	126.43	164.91	125.97	6.6	5.27	0.05	61.30	0.0027
	Median	53.22	7.91	16.9	1147	847	79	37.75	278	121.5	187.5	120.5	6.55	5.06	0.03	34.37	0.002
	Max	161.2	8.1	25.8	8181	1114	150.2	52	399.7	210	243	207	12	10.8	0.18	232.75	0.0067
	Min	6.45	7.31	11.8	92	517	44	9.4	190	91	45	70	3.2	2.2	0.01	11.43	0.001
	Std	41.40	0.20	4.36	2178.71	211.85	28.64	11.05	71.02	30.39	61.53	43.06	2.29	3.09	0.04	72.5	0.001

Wet season	*n*	13	13	8	10	13	13	10	11	11	11	13	13		12	9	13
	Mean	248.4	7.70	25.35	14760.2	469.46	51.67	17.35	149.09	108.71	56.85	48.88	5.69		0.16	26.58	0.0025
	Median	219.07	7.65	27.15	8990.5	436	44	14.5	135	100	32	36	5		0.11	24.93	0.0012
	Max	712.77	8.33	28.2	53960	1168	91	36	376	182	268	131	9.9		0.458	53.78	0.01
	Min	6.65	7.27	17.7	240	170	19	3.4	59	57.6	20	18	3.2		0.026	2.724	0.0009
	Std	195.22	0.27	3.68	17298.53	255.60	23	10.01	87.87	33.07	71.45	31.33	2.38		0.15	18.75	0.002

*dm*		−3.8	1.88	−4.36	−2.49	4.07	3.09	4.38	4.53	1.41	4.04	5.79	1.07		−2.70	1.73	0.14
